# Thinking computationally in translational psychiatry. A commentary on Neville et al. (2024)

**DOI:** 10.3758/s13415-024-01172-1

**Published:** 2024-03-08

**Authors:** Yumeya Yamamori, Oliver J. Robinson

**Affiliations:** 1 Institute of Cognitive Neuroscience, University College London, London, UK; 2 Research Department of Clinical, Educational and Health Psychology, University College London, London, UK

**Keywords:** Translational, Psychiatry, Computational, Validity, Reinforcement learning

## Abstract

There is a growing focus on the computational aspects of psychiatric disorders in humans. This idea also is gaining traction in nonhuman animal studies. Commenting on a new comprehensive overview of the benefits of applying this approach in translational research by Neville et al. (*Cognitive Affective & Behavioral Neuroscience* 1–14, 2024), we discuss the implications for translational model validity within this framework. We argue that thinking computationally in translational psychiatry calls for a change in the way that we evaluate animal models of human psychiatric processes, with a shift in focus towards symptom-producing computations rather than the symptoms themselves. Further, in line with Neville et al.'s adoption of the reinforcement learning framework to model animal behaviour, we illustrate how this approach can be applied beyond simple decision-making paradigms to model more naturalistic behaviours.

## Introduction

A timely primer by Neville et al. (2024) on computational translational psychiatry discusses opportunities for integrating computational approaches more closely into translational psychiatry research. In this commentary, we expand on their ideas by discussing how this approach can help to improve the translational validity of preclinical tests. We also demonstrate an application of the reinforcement learning approach in modelling more ecological measures of symptoms.

## Thinking computationally in translational psychiatry to improve model validity

Can we recreate the complexity of psychiatric disorders, such as anxiety or depression, in animals other than humans? Although this would be immensely valuable for both theoretical and applied research, it is a tough bet. How about behavioural tests in which animals act in ways that we could reasonably interpret as anxiety- or depression-like? This has been the approach of translational psychiatry for the past couple of decades, but its clinical utility has been disappointing, given the limited success rates of clinical trials for novel psychiatric medicines. The reality is that psychiatric medicines, and central nervous system (CNS) drugs more broadly, are significantly more likely to fail at late-stage clinical trials compared with non-CNS drugs, and too often due to lack of *efficacy* (Hyman, 2012). This points to failures of the preclinical (i.e., nonhuman) models of psychiatric disorders used during early stages of drug development in predicting results in the clinic. The verdict may therefore be that we cannot even mimic anxiety- or depression-like symptoms in animals. But what if we didn’t have to?

When assessing the validity of translational models, *construct* validity is a key criterion. This means that translational models should map onto the same construct as in the human disease/function. The target constructs in psychiatry have traditionally been observable symptoms in human patients, and so preclinical models were developed to emulate these symptoms. However, as Neville et al. discuss, psychiatric conditions are more and more understood as computational processes, and there is a growing push to attempt to treat these conditions by targeting their underlying neurocognitive mechanisms, rather than their clinical presentation/observable symptoms. This shift in focus could be mirrored in translational models; the renewed target constructs should be the core *computations* that give rise to symptoms instead of symptom-analogues (Redish et al., 2022).

This change in perspective has important implications for the way that we evaluate model validity. Computational psychiatry assumes that symptoms are a product of disease-relevant computational mechanisms, but the same mechanisms elicited in nonhuman species may not necessarily result in behaviours that resemble human symptoms. Conversely, animal behaviour that is outwardly similar to human symptoms may be a result of entirely different computational mechanisms. Therefore, thinking more computationally, and by extension mechanistically, means reassessing existing translational models to ensure that they are computationally aligned with human psychiatric processes or developing new ones with this explicit intention. This would potentially target our focus only on disease-relevant mechanisms (i.e., signal) over irrelevant ones (noise)—an important step towards improving the predictive validity of preclinical assays and the effectiveness of psychiatric drug development pipelines.

## Using reinforcement learning to understand a broad range of behaviours in nonhuman animal models

Neville et al. (2024) make a strong case for the explanatory role of the reinforcement learning (RL) framework in translational psychiatry. They argue that we should start by examining existing translational assays within this framework. To this end, we recently reverse-translated an animal approach-avoidance reinforcement learning task (the Vogel conflict task) to study anxiety-related avoidance in humans. Applying the RL framework allowed new mechanistic insights into the relationship between anxiety and avoidance (Yamamori et al., 2023). However, a practical challenge with this approach and many RL tasks more broadly is the simplicity of the task, which involves a series of choices between the same two options (e.g., left vs. right lever press). The simplicity of these tasks may make them hypothetically easier to interpret, but training animals, such as rodents, to complete them is usually a long and expensive process. Behavioural tests that rely on more naturalistic and ecological behaviours, such as free exploration of a spatial environment, are much easier to set up and run in animals, which is why such tests like the elevated-plus maze or the open field test are more popular preclinical models of anxiety (Griebel & Holmes, 2013).

**Fig. 1:**
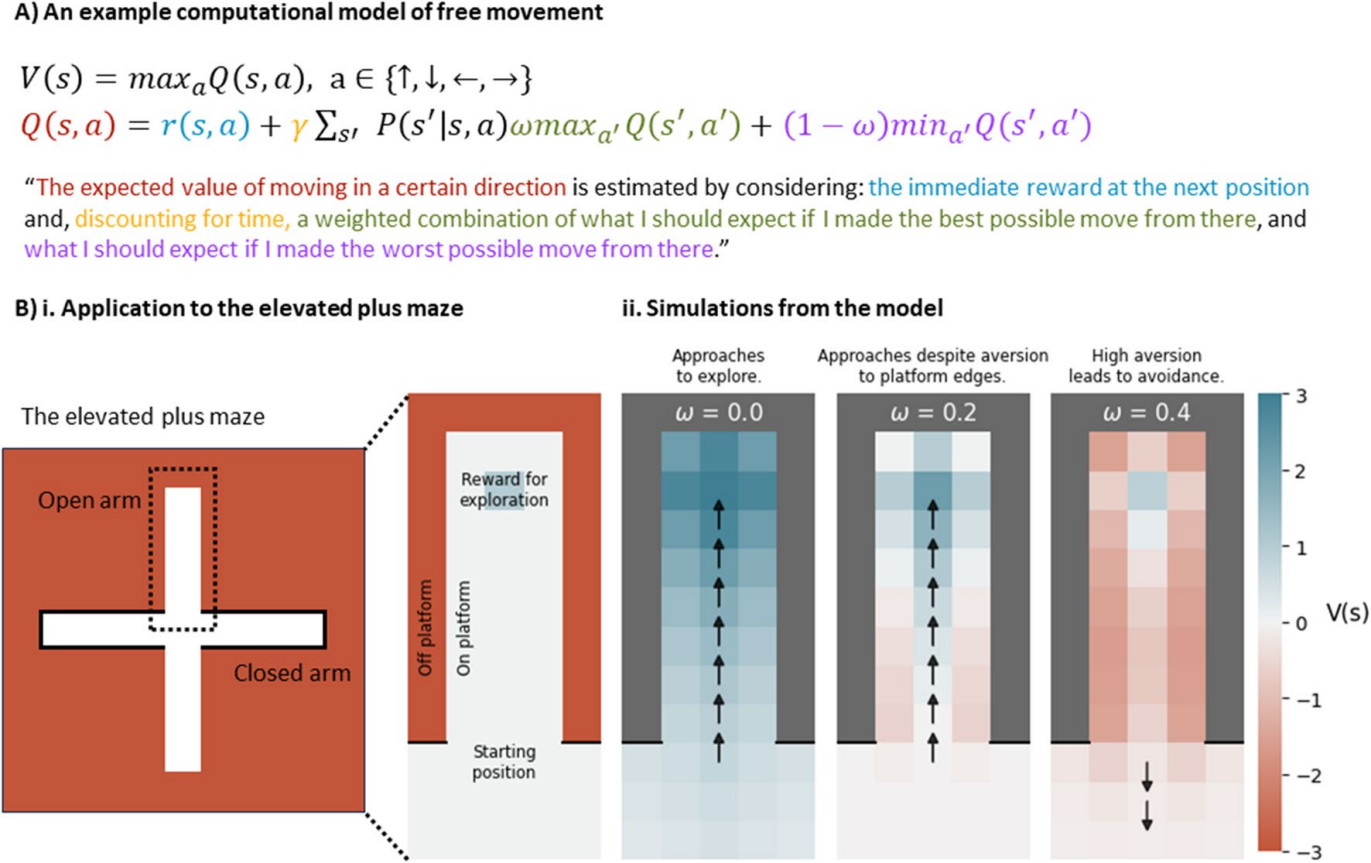
A computational model of two-dimensional movement in the elevated plus maze using a Markov decision process. This demonstrates how pessimism about state transitions can lead to anxiety-like behaviour in the elevated plus maze, when agents are faced with the decision of whether to enter (approach) or avoid the open arm. **A** Model is defined by states of an environment (positions) and a set of actions—up (↑), down (↓), left (←), and right (→), which are evaluated through a value iteration algorithm, incorporating both immediate and anticipated future rewards to calculate the values of states, V(s), and actions from those states Q(s,a). These calculations take into account the best (maximizing Q) and worst (minimizing Q) outcomes, moderated by an avoidance pessimism weight (ω), representing the agent's belief about their ability to avoid future negative outcomes. **B, i.** Applying this model to the elevated plus maze, where subjects move around an environment that involves exposed, open arms and safe, closed arms. **B, ii.** Simulations from the model with differing pessimism parameters. Colours represent the expected value, V(s), of the position on the map, with greater values indicating a greater propensity for the agent to occupy or move toward those states. Arrows represent how each agent would act in each position, beginning from the starting position. The optimistic agent is confident that they will not fall off the platform, so explores the open arm and exhibits low anxiety behaviour. A moderately pessimistic agent shows some fear of the edges of the arm (denoted by negative V(s) near the platform edges) but is still willing to explore the arm. The most pessimistic agent is unwilling to explore the arm, given the potential risk of falling—exhibiting high-anxiety behaviour

Recent work indicates that it also is possible to model this kind of more naturalistic exploration behaviour by using the RL framework by casting it as a *Markov decision process* (MDP). MDPs offer a framework for modelling spatial navigation by conceptualising free exploration as a sequence of interdependent choices. Each choice affects potential future locations and can account for the uncertainty around state-to-state transitions and potential rewards or threats. Drawing on a simulation study by Zorowitz et al. (2020), this approach could be applied to the elevated plus maze (EPM; Fig. 1). Central to this application is the inclusion of an avoidance “pessimism” weight, which represents an agent’s confidence in avoiding future threats. Agents with low pessimism show a greater inclination to explore, even in the open arms of the EPM where subjects can potentially fall off the platform, thus exhibiting “low anxiety” behaviour. As agents become more pessimistic, they value the open arm more negatively, which ultimately results in avoidance because of their low confidence in avoiding falls—an effect that models high anxiety.

This RL-grounded explanation for approach-avoidance behaviours on the EPM then opens up the exploration of multiple different computational mechanisms. For instance, one could manipulate the reward signal for exploration to model exploration (approach) drives or adjust the probability of state transitions to reflect general locomotor activity. As Neville et al. discuss, this would enable the “unmixing” of multiple potential sources of a behavioural readout: for example, to better understand the effect of a novel drug in the elevated plus maze. Such lines of research may offer new insights into the properties of existing translational models.

## Conclusions

Computational approaches to translational psychiatry have the potential to improve translational model validity and therefore contribute to struggling psychiatric drug development pipelines. To this end, there is an abundance of preclinical behavioural tests that are yet to be viewed through a computational lens to determine whether they engage translational computational correlates of psychiatric disease. As Neville et al. note, a practical way forward for the field would be to begin by assessing existing models computationally to better understand their cognitive mechanisms. We propose that this could apply to both simple choice tests but also more naturalistic tests. As the aphorism goes, “All models are wrong, but some are useful.” Whereas current translational models in psychiatry may not capture the full complexity of human disease, by thinking computationally, we may be able to move towards improving their clinical utility.

## Data Availability

Not applicable
